# Tumour Growth Models of Breast Cancer for Evaluating Early Detection—A Summary and a Simulation Study

**DOI:** 10.3390/cancers15030912

**Published:** 2023-01-31

**Authors:** Rickard Strandberg, Linda Abrahamsson, Gabriel Isheden, Keith Humphreys

**Affiliations:** 1 Department of Medical Epidemiology and Biostatistics, Karolinska Institutet, 171 77 Stockholm, Sweden; 2 Center for Primary Health Care Research, Lund University, 205 02 Malmö, Sweden; 3 Intelligent Decisions Analytics AB, 171 65 Solna, Sweden

**Keywords:** breast cancer, early detection, tumour growth, screening, observational study, overdiagnosis, mammography

## Abstract

**Simple Summary:**

Advanced statistical methods can be useful for understanding the roles of breast cancer risk factors in cancer progression and detection, and for assessing impacts of early detection of breast cancer in populations with implemented breast cancer screening programmes. In this article, we summarise approaches for estimating, from observational data, tumour progression models that are inspired by biological arguments. These models have the potential to be used in studies of personalised screening. We describe a simulation study that explores the impact of extending the age of screening invitation, which is currently being considered by Sweden’s National Board of Health and Welfare.

**Abstract:**

With the advent of nationwide mammography screening programmes, a number of natural history models of breast cancers have been developed and used to assess the effects of screening. The first half of this article provides an overview of a class of these models and describes how they can be used to study latent processes of tumour progression from observational data. The second half of the article describes a simulation study which applies a continuous growth model to illustrate how effects of extending the maximum age of the current Swedish screening programme from 74 to 80 can be evaluated. Compared to no screening, the current and extended programmes reduced breast cancer mortality by 18.5% and 21.7%, respectively. The proportion of screen-detected invasive cancers which were overdiagnosed was estimated to be 1.9% in the current programme and 2.9% in the extended programme. With the help of these breast cancer natural history models, we can better understand the latent processes, and better study the effects of breast cancer screening.

## 1. Introduction

The main objective of breast cancer screening programmes is to detect breast cancer tumours early in their progression and thereby increase the chance of treatment being successful. Before national breast cancer screening programmes were introduced widely across Europe and other countries around the globe, several randomised mammography screening trials were conducted. These typically compared a screening/intervention group to a control (no screening) group and used mortality from breast cancer as a primary endpoint. An independent review panel analysed data from nine trials and, using Poisson regression and studying breast cancer mortality, estimated relative risks of approximately 0.8 for invited women aged between 50 and 70 [1,2].

Latent variable model-based approaches—in particular multistate models [3]—have also been used to analyse breast cancer screening trials data, in order to quantify the natural history of breast cancer [3,4]. The simplest multistate model has three states: no detectable cancer, preclinical cancer, and clinical cancer [3]. Transitions between states are not observed, but distributions of time spent in these states can be estimated from observed data, under parametric modelling assumptions. An important quantity in the multistate model is the mean sojourn time, which is the average time spent in the preclinical state. If a woman attends screening when in the preclinical state, there is a probability that her tumour will be detected. The basic 3-state model can be extended in various ways to include additional states.

Since the introduction of population-based screening programmes, a range of (latent variable) model-based approaches have been used to assess the impacts of screening. Notably, in 2000, the US National Cancer Institute established the consortium CISNET (Cancer Intervention and Surveillance Network), which studies breast (and other) cancer mortality trends. For breast cancer, there are six groups [5], each using different models. Some are based on multistate modelling assumptions, while others use continuous growth models.

The CISNET groups used simulation approaches based on calibrations to US population incidence and mortality statistics and employed their natural history models to study the relative impacts of treatment and screening on incidence and mortality trends [6,7]. Changes in these impacts across time are important to study, as both methodologies of screening and breast cancer treatment are continuously evolving [8]. CISNET argues that, by analysing data using several different models, it incorporates model uncertainty. Data on screening attendance were not available in these studies.

It is important to study the impacts of screening not only in terms of early detection and mortality reduction but also in terms of its harms; see Table 2 in Trentham-Dietz et al. [9] for common outputs of the CISNET breast cancer models. Recalling a woman after screening, when further testing does not lead to a diagnosis (i.e., a false positive), can cause a psychological burden [10] and lead to unnecessary resource use. False positives can also reduce the probability that a woman attends further screens [11]. Another major harm of screening is overdiagnosis, which can be defined as screen-detected cancer that would otherwise not have been diagnosed in the woman’s lifetime [12,13].

The age-based screening programmes used in today’s health care systems may, in the future, be replaced by individualised risk-based screening programmes [14], although it must be acknowledged that there are a number of hurdles that first need to be overcome [15]. When designing such programmes, more detailed knowledge of the natural history of breast cancer will be useful. Screening programmes will need to distinguish which women have a high risk of getting the disease but can also make use of individual-level information on how long breast cancers are likely to be present in women’s bodies before symptoms emerge, and on the detectability of the cancers at screening as they progress. In recent years, a number of studies have made use of data on screening attendance collected in population-based studies of breast cancer, in order to learn more about these processes. Screening attendance data can be important for better estimating underlying/latent processes in the natural history of the disease.

In this article, we summarise some recent developments in this area, i.e., for fitting natural history models to observational (and detailed) breast cancer screening data. Our focus is on continuous growth models and invasive breast cancer. Continuous growth models represent a class of models in which tumours are assumed to grow in size according to a particular mathematical growth function. These models have advantages over multistate models in terms of the ease in which individual risk factors can be incorporated and in terms of interpretability. We focus on invasive breast cancer (invasive ductal or invasive lobular) as the models, so far, have not been developed fully for noninvasive breast cancer. After describing the methodology, we provide an example of how continuous growth models can be employed, once trained on observational data. We describe a simulation study which explores consequences of extending the age limit of screening—a potential policy change that has been raised by local political groups and is currently being considered by Sweden’s National Board of Health and Welfare [16]. Our simulation approach does not account for all aspects of the benefits/harms of screening (we do not, e.g., include false-positive results) and should therefore be considered as illustrative. It does, however, show how detailed models of tumour onset, progression, and detection—once trained on observational data—can in turn be used to study impacts of changes in screening policy. Overdiagnosis is a particularly relevant concern to be aware of when considering the consequences of extending the age limit of screening, and this is considered here, albeit only for invasive breast cancer.

## 2. Inference Methods

In this section, we summarise inference approaches that have been proposed for estimating continuous growth models using observational mammography screening data. Just like multistate models, continuous growth models are latent variable models. The simplest continuous growth model assumes that tumour size follows a particular growth function, and it accounts for individual variability between tumours by allowing growth rates to follow a probability distribution. Continuous growth models incorporate biological assumptions more explicitly than multistate models do. Multistate models developed for breast cancer screening data have mostly assumed exponentially distributed transition times in the nonabsorbing states, although other distributional assumptions have also been made [3]. As Uhry et al. [3] have pointed out, the most commonly employed multistate models are prone to underestimating the variances in tumour progression processes. Other problems with “classical” multistate Markov models are described in Section 2.2 of Weedon-Fekjær et al. [17].

### 2.1. Components of Models of Breast Cancer Tumour Progression

The earliest continuous growth model for observational mammography screening data that we are aware of is the one described by Weedon-Fekjær et al. [17,18]. This included two components, namely a growth function for tumour volume (logistic growth function with lognormal random effects) and a logistic screening sensitivity function (of latent tumour diameter). Prior to this, a related approach was described for data collected from unscreened populations, which included components for tumour growth, localised spread, and distant metastatic spread [19]. More recently, a number of models were described for observational screening data, which also incorporate components for symptomatic detection of the primary tumour [20], tumour onset [21], and lymph node metastasis [22]. The inclusion of symptomatic detection of the primary tumour built on an earlier approach described for unscreened populations [23]. The continuous growth model which we will use in our analyses (Section 3) has the following components/submodels:

- *Onset of the primary tumour*—onset is defined as the point at which a tumour reaches a diameter of 0.5 mm (from which it can reasonably be assumed to have deterministic growth), and its distribution is defined according to the Moolgavkar-Venson-Knudson carcinogenesis model [24]. Tumours are assumed to be spherical.
- *Growth of the primary tumour*—tumour volume is assumed to follow an exponential growth function where the inverse growth rate is a gamma random effect.
- *Lymph node spread*—a nonhomogeneous Poisson process with rate of spread assumed to be proportional to the number of cell divisions (raised to a power) and the rate of growth of the primary tumour.
- *Symptomatic detection*—the hazard rate of symptomatic detection is proportional to the (latent) tumour volume.
- *Detection via mammography*—screening test sensitivity follows a logistic function of the (latent) tumour diameter.

The above lymph node spread model has been shown to fit observational data more closely than models based on alternative rate functions (e.g., those that are used in CISNET models) [22]. The exponential growth model (with inverse growth rates distributed as gamma random variables) has convenient mathematical properties: for example, in the absence of screening, the distribution of symptomatic tumour sizes has a closed form. Other choices of the above submodels are of course possible, and theoretical results for general functions of tumour growth and tumour detection have recently been established [25]; see also Section 2.2.1.

### 2.2. Likelihood Inference for Incident Cases and Cohort Designs

Both likelihood [3,4] and Bayesian inference [26] methods have been developed for fitting multistate models to screening cohort data. For trials data, likelihood-based methods have been used to fit multistate Markov models—these have been cohort studies, and likelihoods have, e.g., been constructed on the basis of incidence of interval cancers (modelled using a Poisson process) and the incidence of cancers at screening rounds [3,4,27]. In addition to cohorts, large studies of incident breast cancer have the potential to provide information on latent processes of tumour progression, although these types of studies have rarely been used for this purpose. In this section, we summarise likelihood inference approaches for fitting continuous tumour growth models to both samples of incident cases with information on screening histories and to mammography screening cohort data.

#### 2.2.1. Continuous Growth Models for Collection of Incident Cases

Likelihood inference of tumour progression based on incident cases relies on the concept of a stable disease population, which describes the disease population dynamics of women with breast cancer, under the assumption of no screening. Screening conditions are then imposed on to the stable disease population through the likelihood function. The general idea behind the likelihood is to probabilistically project the tumours backwards in time to the hypothetical times of onset—taking each woman’s screening history, mode of detection, and tumour characteristics into account—and from there, at a population level, to infer the most likely growth trajectories for tumours.

The stable disease population is represented by breast cancer incidence being constant (in the absence of screening) and by the disease progressing according to time-constant rules. In Isheden and Humphreys [28], a stable disease population was formalised in the absence of screening by assuming that each woman can pass through three discrete states:

- P_free_—a disease-free state (prior to breast cancer tumour onset).
- P_tumour_—a breast cancer state (preclinical/as yet undetected).
- P_after_—a post-symptomatic detection state.

A woman can only pass from P_free_ to P_tumour_ and from P_tumour_ to P_after_. Three further assumptions about the population were also made:

- The rate of births in the population is constant across calendar time.
- The distribution of age at tumour onset is constant across calendar time.
- The distribution of time to symptomatic detection is constant across calendar time.

These formulations and assumptions make the connection with multistate Markov models explicit (the same assumptions are in fact made in multistate Markov models). In continuous growth models, however, tumour growth is modelled explicitly on a continuous scale and, unlike multistate models, entrance to P_tumour_ is defined explicitly in terms of a (small) tumour size at which tumours become detectable.

The above three assumptions together lead to a constant rate of new cancers entering the population and a constant incidence of (diagnosed) breast cancers. From these assumptions, two important properties were formalised in mathematical lemmas [25].

The first relates to the probability of screening a woman with breast cancer and states that “The probability for an individual to have a pre-clinical tumour at a particular/current time point is proportional to the time it will spend in tumour growth”.

The second property relates to the time a screen-detected woman has spent in the preclinical state and can be stated as “For an individual currently with a pre-clinical tumour, the probability that tumour onset occurred *t* years earlier is uniformly distributed over the eventual time it will spend in the pre-clinical state”.

These two properties greatly simplify likelihoods for observable data (tumour volumes given mode of detection and history of negative screens) in terms of tumour growth, screening sensitivity, and symptomatic hazard functions. Isheden and Humphreys [25] essentially showed that likelihood inference of tumour progression based on these three functions can be carried out as long as the functions are calculable and the tumour growth function is monotonously increasing (they presented explicit results for exponential, Gompertz, and logistic growth functions). This was made possible by displaying two further properties of the progression models under stable disease assumptions.

The first property is that the probability density function for the growth parameter conditioned on there being an undetected tumour of size *v* is equal to the probability density function for growth rate conditioned on a tumour being symptomatically found at size *v*.

The second property is that the probability density for tumour size, conditioned on an individual belonging to the preclinical tumour growth state, is proportional to the probability density of volume at symptomatic detection divided by the hazard for symptomatic detection at that volume.

Based on the above-mentioned properties and functions, a likelihood for tumour size conditional on screening history, clinical characteristics, and mode of detection can be calculated and used to estimate the parameters of the (e.g., tumour growth) functions, with limited bias.

Finally, we note that the stable disease assumption described here is typically used also in cohort studies (both when fitting multistate and continuous growth models), although there the assumptions are not relied on so explicitly as they are with incident cases.

#### 2.2.2. Continuous Growth Models for Screening Cohorts

The continuous growth model of Weedon-Fekjær et al. [17,18] was fitted to data from a screening examination in the Norwegian Breast Cancer Screening Program. In [18], this was performed by optimising a likelihood function that was based on jointly modelling the incidence of cases at screening and the incidence of interval cancers (the cancers detected symptomatically after the prevalent screen and before the next scheduled screen). In [17], tumour sizes of the screen-detected cases (using a multinomial distribution) and the incidence of interval cancers (using a Poisson distribution) were jointly modelled. Strandberg et al. [29] fitted a continuous growth model to mammography screening cohort data by jointly modelling age at detection, mode of detection (screen/symptomatic), and tumour size, conditional on screening attendance. This was made possible by the inclusion of a submodel for age at tumour onset. This also made it possible to incorporate the left-truncation (women previously diagnosed with breast cancer are not included) inherent in these kinds of cohorts. From an epidemiological methods/statistical epidemiology point of view, these approaches are more conventional than those described above for the collection of incident cases (Section 2.2.1), so we do not provide additional descriptions of the methods here but refer the reader to the original articles. We note that in Strandberg et al. [29], risk of tumour onset was modelled as a function of a number of breast cancer risk factors. There is good reason to believe that the cohort design is likely to be more reliable (more strongly identified parameter estimates, lower variances) than the incident cases design for estimating the parameters of the natural history models, because the cohort approach uses information on time (age at diagnosis) that is not included in the incident cases approach. Strandberg et al. [29] concluded, in their cohort study, that their estimates for growth rates and tumour doubling times were comparable to those obtained from studies of sequential mammograms/ultrasound images.

### 2.3. Simulation-Based and Inference-Based Evaluations of Early Detection

Consequences of early detection of breast cancer can be evaluated in different ways. Screening trials and CISNET simulation approaches focus on the effect of the intervention, i.e., of being invited to screening, or the effect of participating in the programme on breast cancer mortality. This is the type of approach we took in our simulation study (Section 3). It is, however, also possible to study the effect of being screen-detected, e.g., on breast cancer survival. Analysis of trials data for studying the effect of invitation to/participation in screening on breast cancer mortality is relatively straightforward (although consideration may need to be given to adherence to “treatment” or healthy screenee bias [30]) and can be based on estimating relative risks. Focusing on screening participation, CISNET have used simulation-based approaches extensively, e.g., to gain insights into the relative contributions of treatment and screening across time [6,7]. This type of analysis is more complex.

For evaluating the effect of being screen-detected on breast cancer survival, special care has to be taken to handle a number of biases [31] which can have a large impact on survival comparisons between different sets of individuals, in particular between those detected by screening and those detected in the interval between two screens. The biases, which specifically occur in screening data, result from lead time (the time between screen detection and when the tumour would have been detected through symptoms), length time (may be defined as the time a tumour is observable by screening or the time a tumour is present in a woman’s body), and overdiagnosis (women diagnosed with breast cancer by screening, whom in the absence of screening would not have been diagnosed in their lifetime).

Based on the continuous tumour growth model, including separate models for tumour growth, time to symptomatic detection, and mammography screening sensitivity, Abrahamsson et al. [32] derived a formula for the lead-time distribution, conditional on a screen-detected individual’s tumour size at detection, previous screening history, and mammographic percentage density. These distributions are informative in their own right, e.g., for quantifying how lead time is longer for tumours that are small at screen detection. If covariates are included in the different submodels of the continuous tumour growth model as, for example, in Abrahamsson et al. [33], it is possible to make individual-level comparisons. From quantifying the inverse association between breast size and rate of symptomatic detection [33], it is possible to quantify the extent to which lead time is longer for women with large breasts than for women with small breasts. The conditional lead-time distributions can in turn be used to correct for the lead-time bias in survival comparisons, by subtracting lead times from screen-detected cases’ survival times. Lead times may also be useful for estimating an individual’s risk of being overdiagnosed.

Abrahamsson et al. [32] also described a simulation study using the same tumour growth model, to illustrate potential biases and causal effects of screen detection on breast cancer survival. Additional models for the risk of breast cancer (age at tumour onset), death from breast cancer (measured from time of detection), and deaths from causes other than breast cancer were postulated for these comparisons. Different counterfactual scenarios (in terms of screening attendance) were simulated. These simulations were used to study biases of lead time and length time. It was demonstrated that not only the tumour growth rate but also the symptomatic tumour size is a part of the length bias through any link between tumour size and survival, and it was also explained how this has a bearing on the way that observable breast cancer-specific survival curves should be interpreted.

Finally, we note that with continuous growth models, equations can be derived to infer the impacts of screening directly. Isheden et al. [34] demonstrated this for lymph node spread. For lymph node-positive cancers at the time of diagnosis, they characterised the probabilities of having already seeded lymph node metastases at different lengths of time prior to detection of the primary tumour and evaluated, for screen-detected lymph node-negative cancers, the probabilities that they would have exhibited lymph node spread if their primary tumours had instead been detected later in time.

## 3. A Simulation Study—Extending the Age of Screening Participation

A mammography screening programme with nationwide coverage has been in place in Sweden for more than three decades. Currently, women in Sweden are invited to mammography screening between the ages of 40 and 74, every 18–24 months (depending on the healthcare region) [35]. There have been some suggestions from local political groups that the age limit should be increased [16].

To showcase how continuous growth models can be used to assess screening (which can be easily extended to risk-based individual screening), we present a simulation study based on hypothetically extending the current screening programme in Sweden to age 80. This amounts to adding three more screening rounds—at 76, 78, and 80. We use simulations from a breast cancer natural history model to compare the outcomes in a population subjected to such an extended programme to the outcomes of the current programme.

### 3.1. Parameter Values Used in the Simulation

Our simulations are based on models that have previously been estimated on data from a large Swedish mammography screening cohort [29] and estimates of breast cancer survival taken from published literature; see Appendix A and Appendix B. The parameter estimates of the tumour onset, progression, and detection models that we obtained from the Swedish screening cohort are maximum likelihood estimates (see [29] for a description of the estimation procedure). Point estimates and confidence intervals are provided in Table A1 in Appendix A. Estimates of the variance of the parameter estimates were used in some of the simulations. For example, when estimating life expectancy increases due to attending screening, 90% confidence intervals for the means were generated through propagating the estimation errors by sampling parameter values from a multivariate normal distribution using the point estimates as the mean vector and the inverse of minus the hessian of the log-likelihood function as the covariance matrix (see Section 3.3; Results). This was performed over 100 simulations, each containing one million in silico individuals. Uncertainties in estimates of breast cancer survival were not incorporated (uncertainties were unknown since the estimates were extracted from published sources). For other simulations, results are based on averages from ten simulations, each of one million individuals, and do not incorporate variability in parameter estimates (see Section 3.3). For evaluating overdiagnosis, we used external data on mortality from causes other than breast cancer. Mortality rates from causes other than breast cancer were extrapolated by subtracting the 2019 Swedish breast cancer-specific mortality rates from NORDCAN [36] from the 2019 Swedish all-cause mortality rates from population data by Statistics Sweden [37].

### 3.2. Description of the Simulation Approach

We simulated data using the four-component model presented by Strandberg et al. [29]. This consists of the Moolgavkar-Venson-Knudson carcinogenesis model for the age at onset, exponential tumour growth with gamma-distributed inverse growth rates, a continuous hazard of symptomatic detection proportional to the concurrent tumour volume, and logistic functions for the screening sensitivity proportional to the concurrent tumour diameter. Additionally, lymph node metastasis is simulated according to the model developed by Isheden et al. [34]. According to this model, the number of affected lymph nodes at diagnosis, conditional on the observed tumour size, follows a negative binomial distribution. As described in Section 3.1, the submodel formulas and parameter values used are listed in Appendix A.

We begin by sampling the age at onset and the inverse growth rate of the tumour. Based on these, we sample the age when symptomatic detection will occur, including the tumour size and number of affected lymph nodes at detection. We then superimpose a screening programme where the screening sensitivity at each screening round is calculated, and the result is simulated. Screen detection occurs at the first positive screen, with concurrent tumour size and sampled lymph node status. (Note that, since the screening programmes are not indefinite, screen detection is not guaranteed. In those cases, the age is set to infinity, and missing tumour size and node status are omitted).

Based on the outcome of each of the two modes of detection, two separate breast cancer survivals are sampled, along with age of death from other causes. The observed outcome is then determined by whichever occurs first, either screen-detected breast cancer, symptomatic breast cancer, or death before breast cancer diagnosis. For the breast cancer cases, cause of death and all-cause survival is determined by whichever death occurs first.

We then tally the following metrics for each screening programme we simulate:

- Number of mammograms performed.
- Number of breast cancer cases.
- Number of cases detected through screening.
- Number of overdiagnosed cases.
- Stage shift, which occurs when early detection causes the stages of either the primary tumour size or the number of affected lymph nodes to shift down one or more levels according to the categorisation as described in Table 1 (T: primary tumour or N: lymph node metastasis).
- Number of breast cancer deaths.
- Lead time, the time ”gained” between screen detection and would-be symptomatic detection.
- Survival differences (all causes, difference between screen-detected and would-be symptomatic diagnosis).

### 3.3. Results

In Table 2, we compare three different screening programmes: no screening; biennial screening between 40 and 74, representing the current Swedish programme; and biennial screening between 40 and 80, representing a proposed extension to the Swedish programme. Each programme is simulated using the procedures described above, and the numbers presented are averages over 10 runs of one million in silico women each. The columns labelled ”None”, ”Current”, and “Extended” represent these respective screening programmes. We separate the cases into the following five categories, four of which represent screen-detected cases:

- Overdiagnosed—screen-detected cancer but has a non-cancer-related death before the time she would have been symptomatically detected.
- T-shifted—cases that were not overdiagnosed and where the T-stage was shifted due to early detection (but N-stage was not shifted).
- N-shifted—cases that were not overdiagnosed and where the N-stage was shifted. (This category also includes cases which where both T- and N-shifted.)
- Other screen-detected—the remainder of the screen-detected cases.
- Interval cancer—detected symptomatically between the current and next screening rounds.

The 151,376 cases observed under no screening (Table 2) imply a lifetime breast cancer risk of 15.1%. In the current screening programme, 57,608 women are screen-detected with breast cancer, of which 1070 are overdiagnosed. This implies that 0.1% of women will be overdiagnosed with breast cancer under the current programme. The number of breast cancer deaths is reduced by 18.5%, and the average survival difference is 2.79 years.

If screening is extended to age 80, the number of mammograms performed increases by 12.9%, and 24.7% more cases are detected through screening. The number of breast cancer deaths decreases by another 3.9%, but the number of overdiagnosed women increases by 95%. Compared to no screening, the number of breast cancer deaths is reduced by 21.7%.

For the 1301 women whose death from breast cancer was prevented by attending the extended screening programme, survival times increased by a mean (median) of 4.1 (3.1) years. For the 1018 additionally overdiagnosed cases, the mean (median) remaining life years was 3.7 (2.4). Due to the unnecessary treatment, these life years would be of a lower quality compared to not attending the extended programme.

**Figure 1:**
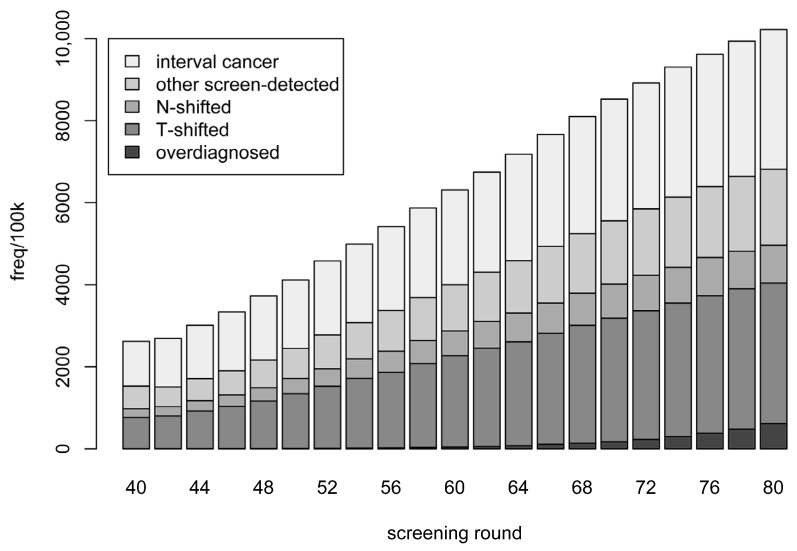
Categories of breast cancer cases detected in relation to each screening round. Categories are defined in the text. Frequencies are stacked and represent averages of ten simulations, each based on one million in silico individuals.

Instead of considering a screening programme in its entirety, we can inspect each screening round separately. In Figure 1, we present a breakdown of the case types for each screening round in the extended screening programme. (Note that the results for each screening round up to age 74 are identical to those that would be obtained when simulating from the current screening programme). Numbers are presented as per 100,000 mammograms performed. The number of cases (per 100,000 mammograms) increases with age—including the number of screen-detected cases. The proportions of screen-detected cases at each screening round which are overdiagnosed ranges from 0.1% to 4.8% over the current screening programme; the respective proportions for the additional screening rounds are 5.9%, 7.2%, and 10.0%.

**Figure 2:**
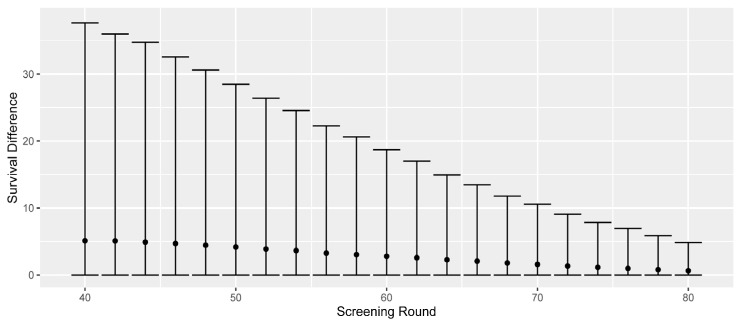
Differences in all-cause survival for screen-detected cases compared to their would-be symptomatic survival. The mean, 5th, and 95th percentiles are separated by screening round of detection. Numbers are based on aggregating ten simulations, each based on one million in silico women.

For the screen-detected cases, we can estimate survival differences that are due to early detection. (For all but the screen-detected cases, this survival difference is zero). In Figure 2, we present the differences in all-cause survival, comparing the observed screen-detected survival to the would-be symptomatic survival. To account for lead-time bias, we begin counting the screen-detected survival from the time of would-be symptomatic detection. The screen-detected cases are separated by screening round, and the mean, 5th, and 95th percentiles of survival difference are presented.

The mean survival difference decreases as the remaining life expectancy (due to deaths from other causes) decreases. The average survival differences are 5.1 years for screen-detected cases at age 40; 4.2 years at age 50; 2.8 years at age 60; and 1.6 years at age 70. The last screening of the current programme at age 74 shows an average survival difference of 1.1 years, and we observe that extending the screening programmes would provide respective mean survival differences of 1.0, 0.8, and 0.6 years for the additional screenings at 76, 78, and 80. Thus, the average survival difference across the whole extended programme would be lower than that of the current one, as seen in Table 2.

**Figure 3:**
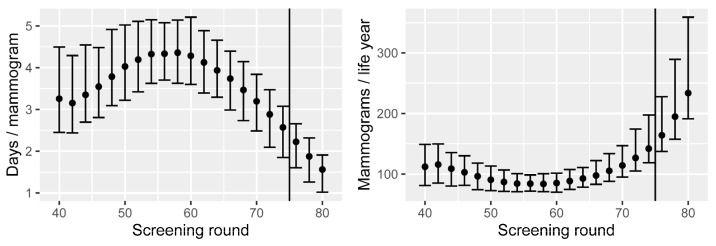
Left: increased life expectancy when attending screening, by screening round, and per mammogram performed. Right: number of mammograms performed per life year gained, by screening round. Means and 90% CIs are based on calibration uncertainty and are calculated as described in Section 3.1.

By adding all the nonzero survival differences for a screening round and dividing by the number of mammograms performed, we obtain the prolonged life expectancy for attending that round. The resulting number is counted in days and is shown in the left panel of Figure 3 for each screening round between 40 and 80.

We observe that the average life expectancy increases by 3.3 days if attending the first round at age 40. This steadily increases until it reaches 4.4 days with the screening round at age 58. The reason for the increase is that breast cancer incidence is relatively low in the 40s. After this, the additional life expectancy decreases until the end of the current screening programme, where the additional life expectancy is 2.6 days if attending at age 74 (Figure 3). The decrease is due to the fact that the remaining life expectancy decreases with age (regardless of the screening). We observe that the extra life expectancy decreases further for each of the three additional screening rounds—2.2, 1.9, and 1.6 days, respectively. The estimated combined life expectancy change for the current screening programme, considered in its entirety, is currently 60.8 days (90% CI: 51.7–71.4), whilst it is 64.8 days (90% CI: 55.0–76.4) for the extended programme.

The reciprocal of these results can be represented as the number of mammograms performed per year of life (life year) gained, seen in the right panel of Figure 3. The average number of mammograms required across the current programme is 101, and each new round from age 76 to 80 would require 164, 195, and 234 mammograms per life year, respectively.

## 4. Discussion

In this article, we first summarised recently developed approaches for inferring processes of tumour progression, based on continuous growth models, from observational breast cancer screening data, and we then presented a simulation study using tumour onset, growth, and spread models—estimated using data from a Swedish screening cohort [29]—together with published survival data to investigate the hypothetical effects of extending the Swedish screening programme to age 80.

In the first part of this article, we summarised approaches that have been developed for both samples of incident cases and for screening cohort designs. Although we described fundamental assumptions of the models and other assumptions upon which estimation procedures are based, it is important to note that other intricacies/assumptions have, to some extent, been overlooked. For example, we did not consider implications of an association between growth rate and screening attendance. In particular, though, samples may in practice be subject to selection processes (e.g., may include only women of screening age), which may have some bearing on the consistency of the parameter estimates. In the Swedish cohort study, we accounted for left truncation. For incident cases, such selection processes can be more difficult to account for. Whilst these are interesting issues from a statistical perspective, they are probably not crucially important, and it has to be acknowledged that these issues have also been omitted in the considerable literature on more conventional multistate Markov models. The statistical analysis of cancer screening data is notoriously complex, with many subtle issues and sources of bias.

Our simulation study illustrates how natural history models can be used for studying screening. However, it is important to note that we have combined models based on data from different countries (the survival models were not trained on Swedish data). Some caution should therefore be taken when interpreting results. In our study—investigating extending the age of screening invitation—we could observe how additional screening rounds increase early detection; the 13% more mammograms allowed early diagnosis of 25% more cases. Not surprisingly, screening at later ages considerably increases the rate of overdiagnosis: the rate is nearly doubled compared to the current programme. For policy makers, an important question that is related to other questions of cost-effectiveness [38] is whether or not such absolute rates of overdiagnosis would be tolerable. We also observed (results not shown) that overdiagnosis rates increase rapidly if the upper age of screening is extended even further. Additional screenings will further reduce breast cancer mortality, but survival differences will be lower than at previous screening rounds, due to lower conditional life expectancies. While further considerations of the benefits and harms are necessary for any decision to be made, our simulation study demonstrates key points and illustrates how effects of changing screening can be quantified. These kinds of results could, in principle, be used to inform the design of a screening trial. With regard to harms of screening, a particular limitation of our simulation, as mentioned in the Introduction, is that we do not include false positives. To include false positives, we would require a model of screening specificity. CISNET uses specificities of film and digital mammography provided by the U.S. Breast Cancer Surveillance Consortium as a common input—these values are based on age, breast density, and screening interval (first screen or subsequent, and if subsequent, whether they have annual, biennial, or triennial screening) [39].

Our particular simulation study of screening programmes was strictly age-based, but still demonstrated how screening changes affect population outcomes. The models used—and the simulation study performed—can be extended to individualised risk-based screening. The key to such studies would be to enable each submodel to depend on individual covariates. This is where the observational studies play an important role. For example, Abrahamsson et al. [33] allowed screening sensitivity to depend on mammographic/breast density, tumour growth rates to depend on BMI, and rate of symptomatic detection to depend on breast size. In Isheden et al. [34], both the tumour growth rates and rates of lymph node metastasis were modelled as a function of hormone replacement therapy. This modelling flexibility allow us to untangle various relationships between risk factors and screening. For example, breast density is known to increase breast cancer risk as well as reduce the screening sensitivity by masking. Risk-based screening (screening high-risk individuals more frequently or with particular imaging modalities) will rely on risk prediction tools. For effective risk-based screening, one challenge is to predict the risk of different subtypes of cancer since different subtypes defined, e.g., by gene expressions, have very different prognoses [40,41].

A limitation of our simulation study is that it only considers invasive cancers. Ductal carcinoma in situ (DCIS) is a precursor to invasive breast cancer and accounts for approximately 20% of all breast cancers. Detected cases of DCIS are usually considered to have limited malignant potential (while DCIS with high malignant potential will typically have progressed to invasive by the time it is detected). The DCIS cases which are detected are predominantly screen-detected. This combination of features means that DCIS most likely constitutes the majority of alleged overdiagnosis [13], and when DCIS cases are included, estimates of levels of overdiagnosis are much higher than those reported here [42]. At the same time, screen-detected DCIS represents the ideal outcome for early detection, provided that it would progress to invasive and not be a case of overdiagnosis. To obtain the full picture of mammography screening, DCIS needs to be better understood in order to separate the screen-detected cases into cases of successful early detection and cases of overdiagnosis.

## 5. Conclusions

Natural history models can be useful for understanding the underlying events and processes of breast cancer and represent useful tools for assessing screening, particularly when programmes are already implemented and widespread. We summarised recent developments for estimating one type of these models, continuous growth models from observational screening studies. If these models can capture the complex roles of breast cancer risk factors in cancer progression and detection, they may be able to facilitate the process of designing personalised risk-based screening. Simulation studies using detailed models of tumour onset and progression can be powerful tools for evaluating screening policy changes and risk-based screening, but they need to consider a range of benefits and harms of screening.

## Figures and Tables

**Table 1:** Definitions of tumour stages and stage shift used in the simulation.

Stage	0	1	2	3
T-stage (mm)	-	1–20	20–50	>50
N-stage	0	1–3	4–9	>9

N-stage = lymph node metastasis stage.

**Table 2:** Comparison of outcomes between screening programmes—first between current screening (40–74 biennial) and no screening, then between extended screening (40–80 biennial) and current screening. Number of mammograms, cases, and deaths are presented as numbers per million women.

	No Screening	Current	Change	Extended	Change
Mammograms (k)	0	16,430		18,553	+12.9%
Total cases	151,376	152,446	+0.7%	153,464	+0.7%
Symptomatic/Interval	151,376	46,640		21,678	−46.5%
Overdiagnosed	0	1070		2088	+95.1%
N-shifted	0	8686		10,528	+21.2%
T-shifted	0	38,442		47,362	+23.2%
Other screen-detected	0	57,608		71,808	+24.7%
Breast cancer deaths	41,182	33,550	−18.5%	32,249	−3.9%
Avg. lead time (yrs)	0	2.18		2.21	+1.4%
Avg. surv. diff. (yrs)	0	2.79		2.37	−15.1%

**Figure A1:**
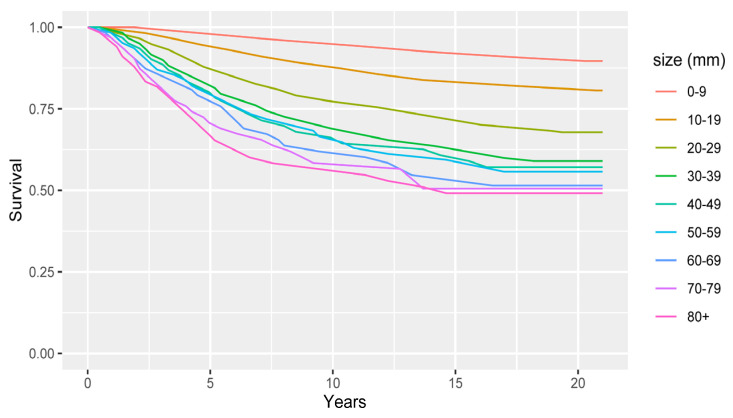
Kaplan-Meier curves for lymph node-negative (N0) breast cancer survival, stratified by primary tumour size.

## Data Availability

The data presented in this article are available on request from the corresponding author. The data are not publicly available due to being large, simulated data sets with up to 100 different runs. The description of the simulation procedures and the contents of the appendices should be sufficient to understand how the data was generated. Upon request, one simulated example data set can be provided.

## Appendix A. Submodels and Parameter Values

The Moolgavkar-Venson-Knudson model used for the age at onset is given by the survival function

$$G_{T}\left(t\right)=P\left(T>t\right)={\left[\frac{\left(B-A\right)e^{Bt}}{Be^{\left(B-A\right)t}-A}\right]}^{\delta },$$

with the three parameters A<0, B>0, δ>0.

The tumour growth is exponential with function $V\left(x\right)=v_{0}e^{\frac{x-t}{r}}$, where $v_{0}\approx 0.0655$ mm^3^, and where the inverse growth rate r comes from the gamma distribution with probability density function

$$f_{R}\left(r\right)=\frac{1}{r\Gamma \left(\frac{1}{\phi }\right)}{\left(\frac{r}{\mu \phi }\right)}^{\frac{1}{\phi }}\exp\left(-\frac{r}{\mu \phi }\right),$$

with the paramters μ, ϕ>0, where E[R]=μ, $\mathrm{Var}\left(R\right)=\phi \mu^{2}$.

The rate of symptomatic detection is given by the continuous hazard function

$$P\left(U^{\prime }\in \left(z,z+\Delta z\right]|U^{\prime }>z\right)=\eta V\left(z\right)\Delta z+o\left(\Delta z\right),$$

where *V*(*z*) is the concurrent latent tumour volume at time *z*.

The screening sensitivity is determined by the logistic function

$$STS\left(d,m\right)=\frac{\exp(\beta_{0}+\beta_{s}d)}{1+\exp(\beta_{0}+\beta_{s}d)}.$$

for parameters $\beta_{0},\;\beta_{s}$, where *d* is the concurrent latent tumour diameter.

The number of affected lymph nodes *N* at detection, given the primary tumour volume V=v at detection, is distributed according to a negative binomial with probability mass function

$$P\left(N=n|V=v\right)=\left(\begin{array}{c} n+\gamma_{1}-1\\ n \end{array}\right)\frac{\gamma_{2}^{\gamma_{1}}\;{\left(\ln{\left(\frac{v}{v_{0}}\right)}^{k+1}\right)}^{n}}{{\left(\ln{\left(\frac{v}{v_{0}}\right)}^{k+1}+\gamma_{2}\right)}^{\gamma_{1}+n}},$$

with the parameters $k,\;\gamma_{1},\;\gamma_{2}>0$, and $v_{0}\approx 0.0655\;{\mathrm{mm}}^{3}$. This has been shown to be consistent with a model in which lymph node spread follows a nonhomogeneous Poisson process with rate of spread proportional to the number of cell divisions (raised to a power, *k*) and the rate of growth of the primary tumour [22].

The values of the parameters of each respective submodel used in the simulations (with 95% confidence intervals, which were used to construct the error bars in Figure 3) are presented in Table A1.

**Table A1:** Parameter values used in the simulation study.

Parameter	Estimate	2.5%	97.5%
*A*	−0.0679	−0.0329	−0.1402
*B*	0.0017	0.0009	0.0033
*δ*	0.0990	0.0242	0.4055
*µ*	0.9706	0.7557	1.2467
*φ*	0.5266	0.4304	0.6443
ln(*η*)	−8.8015	−8.9698	−8.6333
*β*_0_	−4.8344	−5.2303	−4.4386
*β_s_*	0.4948	0.4391	0.5504
*k*	1.9526	1.3950	2.7330
ln(*γ*_1_)	−1.4237	−1.5807	−1.2667
ln(*γ*_2_)	5.7300	4.1528	7.3070

## Appendix B. Survival Functions

In the simulation case study, we used breast cancer survival functions taken from a Surveillance Epidemiology and End Results (SEER) program study [43]. The Kaplan-Meier curves for lymph node-negative (N0) breast cancer survival, stratified by tumour size, are plotted in Figure A1.

The baseline hazards in Figure A1 were modified by (proportional) hazard ratios based on the N-stage at diagnosis [44]. These are shown in Table A2.

**Table A2:** Hazard ratios used to define survival rates in the simulation.

N-Stage	N0	N1	N2	N3
Hazard ratio	1	1.35	2.19	3.48

## References

[1] The Independent UK Panel on Breast Cancer Screening (2012). The benefits and harms of breast cancer screening: An independent review. Lancet.

[2] Marmot M.G., Altman D.G., Cameron D.A., Dewar J.A., Thompson S.G., Wilcox M. (2013). The benefits and harms of breast cancer screening: An independent review. Br. J. Cancer.

[3] Uhry Z., Hédelin G., Colonna M., Asselain B., Arveux P., Rogel A., Exbrayat C., Guldenfels C., Courtial I., Soler-Michel P. (2010). Multi-state Markov models in cancer screening evaluation: A brief review and case study. Stat. Methods Med. Res..

[4] Duffy S.W., Chen H.-H., Tabar L., Day N.E. (1995). Estimation of mean sojourn time in breast cancer screening using a Markov chain model of both entry to and exit from the preclinical detectable phase. Stat. Med..

[5] Alagoz O., Berry D.A., de Koning H.J., Feuer E.J., Lee S.J., Plevritis S.K., Schechter C.B., Stout N.K., Trentham-Dietz A., Mandelblatt J.S. (2018). Introduction to the Cancer Intervention and Surveillance Modeling Network (CISNET) Breast Cancer Models. Med. Decis. Mak..

[6] Berry D.A., Cronin K.A., Plevritis S.K., Fryback D.G., Clarke L., Zelen M., Mandelblatt J.S., Yakovlev A.Y., Habbema J.D.F., Feuer E.J. (2005). Effect of Screening and Adjuvant Therapy on Mortality from Breast Cancer. N. Engl. J. Med..

[7] Plevritis S.K., Munoz D., Kurian A.W., Stout N.K., Alagoz O., Near A.M., Lee S.J., Broek J.J.V.D., Huang X., Schechter C.B. (2018). Association of Screening and Treatment With Breast Cancer Mortality by Molecular Subtype in US Women, 2000–2012. JAMA.

[8] Cristiansen S.R., Autier P., Stovring H. (2022). Change in effectiveness of mammography screening with decreasing breast cancer mortality: A population-based study. Eur. J. Public Health.

[9] Trentham-Dietz A., Alagoz O., Chapman C., Huang X., Jayasekera J., van Ravesteyn N.T., Lee S.J., Schechter C.B., Yeh J.M., Plevritis S.K. (2021). Reflecting on 20 years of breast cancer modeling in CISNET: Recommendations for future cancer systems modeling efforts. PLoS Comput. Biol..

[10] Brewer N.T., Salz T., Lillie S.E. (2007). Systematic review: The long-term effects of false-positive mammograms. Ann. Intern. Med..

[11] Román R., Sala M., De la Vega M., Natal C., Galceran J., Gonzalez-Roman I., Baroja A., Zubizarreta R., Ascunce N., Salas D. (2011). Effect of false- positives and women’s char-acteristics on long-term adherence to breast cancer screening. Breast Cancer Res. Treat..

[12] Gunsoy N.B., Garciaclosas M., Moss S.M. (2014). Estimating breast cancer mortality reduction and overdiagnosis due to screening for different strategies in the United Kingdom. Br. J. Cancer.

[13] Ryser M.D., Lange J., Inoue L.Y., O’Meara E.S., Gard C., Miglioretti D.L., Bulliard J.-L., Brouwer A.F., Hwang E.S., Etzioni R.B. (2022). Estimation of Breast Cancer Overdiagnosis in a U.S. Breast Screening Cohort. Ann. Intern. Med..

[14] Clift A.K., Dodwell D., Lord S., Petrou S., Brady S.M., Collins G.S., Hippisley-Cox J. (2021). The current status of risk-stratified breast screening. Br. J. Cancer.

[15] Waters E.A., Taber J.M., McQueen A., Housten A.J., Studts J.L., Scherer L.D. (2020). Translating Cancer Risk Prediction Models into Personalized Cancer Risk Assessment Tools: Stumbling Blocks and Strategies for Success. Cancer Epidemiol. Biomark. Prev..

[16] The Age Limit of Mammography is Being Investigated by the National Board of Health and Welfare. https://www.dn.se/sverige/aldersgrans-for-mammografi-utreds-av-socialstyrelsen/.

[17] Weedon-Fekjær H., Tretli S., Aalen O.O. (2010). Estimating screening test sensitivity and tumour progression using tumour size and time since previous screening. Stat. Meth. Med. Res..

[18] Weedon-Fekjaer H., Lindqvist B.H., Vatten L.J., Aalen O.O., Tretli S. (2008). Breast cancer tumor growth estimated through mammography screening data. Breast Cancer Res..

[19] Plevritis S.K., Salzman P., Sigal B.M., Glynn P.W. (2007). A natural history model of stage progression applied to breast cancer. Stat. Med..

[20] Abrahamsson L., Humphreys K. (2016). A statistical model of breast cancer tumour growth with estimation of screening sensitivity as a function of mammographic density. Stat. Meth. Med. Res..

[21] Strandberg J.R., Humphreys K. (2019). Statistical models of tumour onset and growth for modern breast cancer screening cohorts. Math. Biosci..

[22] Isheden G., Abrahamsson L., Andersson T.M.-L., Czene K., Humphreys K. (2019). Joint models of tumour size and lymph node spread for incident breast cancer cases in the presence of screening. Stat. Methods Med. Res..

[23] Bartoszyński R., Edler L., Hanin L., Kopp-Schneider A., Pavlova L., Tsodikov A., Zorin A., Yakovlev A.Y. (2001). Modeling cancer detection: Tumor size as a source of information on unobservable stages of carcinogenesis. Math. Biosci..

[24] Heidenreich W.F., Luebeck E.G., Moolgavkaar S.H. (1997). Some properties of the hazard function of the two-mutational clonal expansion model. Risk Anal..

[25] Isheden G., Humphreys K. (2022). A unifying framework for continuous tumour growth modelling of breast cancer screening data. Math. Biosci..

[26] Tan K.H.X., Simonella L., Wee H.L., Roellin A.A., Lim Y.-W., Lim W.-Y., Chia K.S., Hartman M., Cook A.R. (2013). Quantifying the natural history of breast cancer. Br. J. Cancer.

[27] Prevost T.C., Launoy G., Duffy S.W., Chen H.-H. (1998). Estimating Sensitivity and Sojourn Time in Screening for Colorectal Cancer: A Comparison of Statistical Approaches. Am. J. Epidemiol..

[28] Isheden G., Humphreys K. (2019). Modeling breast cancer tumour growth for a stable disease population. Stat. Meth. Med. Res..

[29] Strandberg R., Czene K., Eriksson M., Hall P., Humphreys K. (2022). Estimating Distributions of Breast Cancer Onset and Growth in a Swedish Mammography Screening Cohort. Cancer Epidemiol. Biomark. Prev..

[30] Weiss N.S., Rossing M.A. (1996). Healthy Screenee Bias in Epidemiologic Studies of Cancer Incidence. Epidemiology.

[31] Cox B., Sneid M.J. (2013). Bias in breast cancer research in the screening era. Breast.

[32] Abrahamsson L., Isheden G., Czene K., Humphreys K. (2019). Continuous tumour growth models, lead time estimation and length bias in breast cancer screening studies. Stat. Methods Med. Res..

[33] Abrahamsson L., Czene K., Hall P., Humphreys K. (2015). Breast cancer tumour growth modelling for studying the association of body size with tumour growth rate and symptomatic detection using case-control data. Breast Cancer Res..

[34] Isheden G., Czene K., Humphreys K. (2021). Random effects models of lymph node metastases in breast cancer: Quantifying the roles of covariates and screening using a continuous growth model. Biometrics.

[35] Olsson S., Andersson I., Karlberg I., Bjurstam N., Frodis E., Håkansson S. (2000). Implementation of service screening with mammog-raphy in Sweden: From pilot study to nationwide programme. J. Med. Screen..

[36] Association of the Nordic Cancer Registries NORDCAN. https://nordcan.iarc.fr/.

[37] The Statistical Database of Statistics Sweden. https://www.statistikdatabasen.scb.se/sq/133506.

[38] Alam Khan S., Hernandez-Villafuerte K.V., Muchadeyi M.T., Schlander M. (2021). Cost-effectiveness of risk-based breast cancer screening: A systematic review. Int. J. Cancer.

[39] Mandelblatt J.S., Near A.M., Miglioretti D.L., Munoz D., Sprague B.L., Trentham-Dietz A., Gangnon R., Kurian A.W., Weedon-Fekjaer H., Cronin K.A. (2018). Common Model Inputs Used in CISNET Collaborative Breast Cancer Modeling. Med. Decis. Mak..

[40] Sørlie T., Perou C.M., Tibshirani R., Aas T., Geisler S., Johnsen H., Hastie T., Eisen M.B., van de Rijn M., Jeffrey S.S. (2001). Gene ex-pression patterns of breast carcinomas distinguish tumor subclasses with clinical implications. Proc. Natl. Acad. Sci. USA.

[41] Wu C., Zhou F., Ren J., Li X., Jiang Y., Ma S. (2019). A Selective Review of Multi-Level Omics Data Integration Using Variable Selection. Biotech.

[42] Chootipongchaivat S., van Ravesteyn N.T., Li X., Huang H., Weedon-Fekjær H., Ryser M.D., Weaver D.L., Burnside E.S., Heckman-Stoddard B.M., de Koning H.J. (2020). Modeling the natural history of ductal carcinoma in situ based on pop-ulation data. Breast Cancer Res..

[43] Zheng Y.-Z., Wang L., Hu X., Shao Z.-M. (2015). Effect of tumor size on breast cancer-specific survival stratified by joint hormone receptor status in a SEER population-based study. Oncotarget.

[44] Saadatmand S., Bretveld R., Siesling S., Tilanus-Linthorst M.M.A. (2015). Influence of tumour stage at breast cancer detection on survival in modern times: Population based study in 173,797 patients. BMJ.

